# An Epidemic Experiment

**Published:** 1920-08-28

**Authors:** 


### An Epidemic Experiment.

An experiment during a measles epidemic is referred to by Dr. Joseph Cates in his 1919 report as Medical Officer of Health for St. Helens. More than the usual importance attaches to the course of this outbreak, he says, because for the first time "contacts" were allowed to attend school. For many years it had been the practice in St. Helens rigorously to exclude from school all children coming from a house in which infectious disease occurred. In opposition to this action there were those who considered any benefit accruing from school exclusion was largely diminished by the opportunities for infection arising from the association of the children in the home, at play, and in places of amusement. The Medical Officer of Health decided to try the experiment of permitting all contacts to attend school under strict nursing supervision. The staff of health visitors was increased, so that the borough might be divided up into twenty districts, each containing one or more schools, with a total of about a thousand children in attendance. Arrangements were then made so that every child in each class could be rapidly surveyed by a nurse during the morning session, and particular care bestowed on children known to be in contact with infectious disease. This daily visiting of each class made it easy to send home any child who seemed to be ailing, and also enabled the Medical Officer to keep more closely in touch with any attempt of the disease to gain an entrance into the school. At no time, indeed, did the epidemic appear to spread through the agency of the schools. To the thoroughness of the nursing supervision must be attributed the success of the experiment.

